# Factors associated with in-hospital mortality after stroke at a tertiary hospital in Angola

**DOI:** 10.3389/fneur.2026.1944436

**Published:** 2026-10-19

**Authors:** Sergio Neto, Benvindo Cuenda, Maria Ngueve Major, Jose Kapele, Cliofas Pinto

**Affiliations:** 1 Neurosurgery Service, Clinica Girassol, Luanda, Angola; 2 Neurosurgery Service, Hospital do Prenda, Luanda, Angola

**Keywords:** administrative data, Angola, in-hospital mortality, length of stay, stroke, Sub-Saharan Africa

## Abstract

**Background:**

Most Angolan patients with stroke reach hospital beyond the thrombolysis window, and many deaths occur within 72 h of admission. We investigated routinely recorded variables associated with in-hospital mortality at Hospital do Prenda.

**Methods:**

We conducted a single-centre retrospective cohort study at Hospital do Prenda, a public tertiary hospital in Luanda, from January 2023 to December 2025. The unit of analysis was the hospitalisation episode; four episodes ending in transfer were excluded. Hypertension and type 2 diabetes were extracted from free-text fields using predefined string rules. Univariable analyses used Pearson chi-square, crude odds ratios (OR; 95% CI), and Mann–Whitney tests. Exploratory multivariable logistic regression adjusted for age, sex, year, stroke subtype, recorded comorbidities, and head-CT status. Given the low events-per-variable ratio (~7) and poor calibration in some models, adjusted estimates were considered exploratory associations rather than independent prognostic effects.

**Results:**

Among 479 episodes, 75 patients died (15.7%; 95% CI 12.7–19.2). Median age was 58.5 years among decedents versus 52.0 among survivors (*p* = 0.049). Median length of stay was 3 versus 6 days (*p* < 0.0001), reflecting early mortality rather than a prognostic exposure. Mortality increased from 6.7% at age 45–54 years to 28.0% at ≥75 years and remained graded after adjustment (≥75 years: adjusted OR 5.92, 95% CI 2.28–15.38; *p* = 0.0003). Mortality among patients <45 years was also elevated (17.2%; adjusted OR 2.97, 95% CI 1.28–6.87), predominantly among haemorrhagic strokes (22.2% versus 10.8% for ischaemic stroke; age-by-subtype interaction *p* = 0.0009). Sex, year, stroke subtype, recorded hypertension, and diabetes were not associated with mortality. Absence of head CT showed higher crude mortality (30.0% versus 15.0%; OR 2.42, 95% CI 0.90–6.52). Recorded hypertension prevalence was only 19.8%, indicating substantial under-ascertainment.

**Conclusion:**

Older age was the strongest admission-available correlate of in-hospital mortality, while elevated mortality among younger patients was concentrated in haemorrhagic stroke. Under-ascertainment limits interpretation of comorbidities. Absence of stroke-severity, functional-status, ICU, ventilation, and reperfusion data further limits causal or independent prognostic inference.

## 1 Introduction

Stroke is the second-leading cause of adult death worldwide and the third-leading cause of adult disability. The Global Burden of Disease 2019 study placed stroke at roughly 12% of all deaths and over 100 million disability-adjusted life-years lost in that year, with most of the absolute increase since 1990 occurring in low- and middle-income countries (1). In sub-Saharan Africa the case-mix differs from high-income settings: patients are typically younger, the proportion of haemorrhagic stroke is higher in many series, and pre-hospital delay routinely pushes arrival well past the 4.5-h thrombolysis window (2–5). Forecasts expect this gap to widen rather than narrow through 2050, yet the prognostic models most clinicians reach for - iScore, PLAN, the Weimar NIHSS-derived score - were built on North American, European and Australasian cohorts whose case-mix and care pathways are not those of Luanda (6–10).

Two recent Angolan studies describe the local picture in enough detail to set the question this manuscript addresses. Goncalves and Ricardo reported a cross-sectional profile of 139 adults admitted to the neurology service at Clinica Sagrada Esperanca in Luanda between October 2018 and March 2022: median age 59.5, 66% male, hypertension present in 96% of participants, ischaemic stroke in 73.4% of cases, small-vessel disease the leading ischaemic aetiology at 63.6%, median onset-to-door time 14 h, and only 10.8% of patients arriving within 3 h (11). The INTERSTROKE Angola multicentre case–control study (October 2022 through December 2023; four tertiary hospitals in Luanda; 157 cases, 157 age-matched controls) found that hypertension, current smoking, abdominal adiposity, sedentary lifestyle, diabetes, alcohol intake, and elevated BMI all discriminated cases from controls at *p* < 0.01; only 27.4% of stroke cases achieved a favourable Glasgow Outcome Scale of 5 at discharge, and only 38.2% had a favourable modified Rankin Scale of 0–3 1 month after discharge (12). Both papers describe a population that is younger than international norms, arrives late, and carries a heavy modifiable risk-factor burden. Neither addresses what determines in-hospital death within a defined single-centre cohort.

The stroke registry from Hospital do Prenda was assembled for clinical administration, not for primary research. It captures sex, age, admission year, length of stay, discharge diagnosis, and a one-line free-text comorbidity note for each admission. It does not capture the NIH Stroke Scale (NIHSS), the Glasgow Coma Scale (GCS) on admission, the modified Rankin Scale (mRS) before the event, admission glucose or imaging features beyond the broad subtype label, ICU admission, mechanical ventilation, or whether thrombolysis was offered. The absence of these key prognostic variables is a substantial source of residual confounding; the analyses that follow are therefore framed as descriptive associations of registry-captured variables with in-hospital death, not as estimates of independent prognostic effect.

## 2 Methods

### 2.1 Setting

Hospital do Prenda is a public tertiary hospital on the eastern edge of Luanda. It receives neurovascular referrals from the surrounding municipalities but has no dedicated stroke unit and no dedicated neuro-intensive care unit. Around-the-clock computed tomography (CT) is available; continuous MRI coverage is not. During the study period 20 of the 479 analysed episodes (4.2%) had no CT performed. Decisions about critical care for severely affected stroke patients are taken jointly by the internal medicine, neurology and neurosurgery services.

### 2.2 Period, cohort and data provenance

All admissions between 1 January 2023 and 31 December 2025 with a discharge diagnosis of stroke were eligible. The unit of analysis is the hospitalisation episode; the registry does not carry a stable patient identifier, so repeat admissions cannot be joined to a single individual. Four episodes were coded as transferred rather than discharged alive or dead and were excluded from the mortality comparisons; the remaining 479 episodes form the analytic cohort. An anonymised copy (patient names removed) is provided as Supplementary material together with the complete analysis code, so that every result in this manuscript is reproducible from the primary data (Supplementary material S1–S3; verification in Supplementary Table S1).

### 2.3 Variables and their source in the registry

Sex (female/male; 3 episodes with sex not recorded), admission year (2023, 2024, 2025), age in years at admission (12 episodes missing, 2.5%), and length of stay in days are administrative fields with low missingness. Length of stay was taken from the registry field and validated against the admission and discharge dates. Head-CT status (performed/not performed; 459/479, 95.8%), the aetiological sub-diagnosis recorded at discharge (e.g., thrombotic, cardioembolic, intraparenchymal, subarachnoid), the readmission flag (13 episodes) and surgical intervention (none recorded in the entire cohort) are reported descriptively and, for CT, entered in the adjusted analysis.

Stroke subtype (ischaemic, haemorrhagic, unclassified) is the discharge-coding field. After the data-quality audit, 219 episodes (45.7%) were ischaemic, 235 (49.1%) haemorrhagic, and 25 (5.2%) were unclassified (combined from two registry codes, “AVC nao especificado” and the residual category); we report that proportion rather than reclassifying retrospectively.

Comorbidities (hypertension and type 2 diabetes) come from a single free-text line on the discharge summary, not from a coded problem list. Extraction followed an explicit string rule, published verbatim in the analysis code: a token (a comma-, slash- or plus-separated fragment) was coded as hypertension when equal to HTA or HAS or starting with HTA (e.g., “HTA ESSENCIAL”, “HTA CRONICA”); as diabetes when starting with DM or equal to DIABETES or DIABETES MELLITUS. When neither the diagnosis nor its negation could be confirmed, the episode was coded as not recorded and excluded from the relevant denominator rather than treated as a negative. No natural language processing was used. The comorbidity field was non-empty in 158/479 episodes (33.0%); recorded hypertension prevalence was 19.8% (95/479) and recorded diabetes 10.6% (51/479). Because the field is free-text and often blank, misclassification is expected to be non-differential and to bias comorbidity associations toward the null; this is an important limit on interpreting the hypertension and diabetes estimates (Section 4.4).

The registry does not capture the NIHSS, the GCS, the pre-event mRS, admission glucose or laboratory values, ICU admission, mechanical ventilation, or acute reperfusion therapy; these cannot be reconstructed retrospectively and their absence is treated as a boundary of inference throughout.

### 2.4 Outcome

In-hospital death, taken from the discharge field of the registry (alive vs. dead; transferred excluded from the mortality analysis as above).

### 2.5 Statistical analysis

Pre-specified univariable comparisons were retained unchanged. Pearson’s chi-square test (two-sided, uncorrected) was used for categorical variables, with crude odds ratios and Woolf 95% confidence intervals; continuous age and length of stay were compared by the Mann–Whitney test. The mortality proportion is reported with a Clopper-Pearson 95% confidence interval.

Multivariable analyses were exploratory. Three logistic regression models were fitted: Model A (complete case, *n* = 467) with age (continuous), sex, admission year, stroke subtype, recorded hypertension, recorded diabetes and head-CT status entered simultaneously; Model B (*n* = 467), identical without CT status; and Model C (*n* = 479) using age bands with an explicit missing-age category, sex, year, subtype and recorded comorbidity. Odds ratios are shown per 10 years for continuous age. Events-per-variable was approximately 7 in Model A; the Hosmer–Lemeshow test was significant in the smaller complete-case models (*p* = 0.003), indicating poor calibration. Model C, used for the age-band results and shown in Figure 1, reached a c-statistic of 0.649 with Hosmer-Lemeshow *p* = 0.797. All adjusted estimates therefore describe associations rather than independent prognostic effects and should be interpreted with caution; coefficients affected by quasi-separation (the 20 CT-negative episodes) are reported only descriptively.

**Figure 1:**
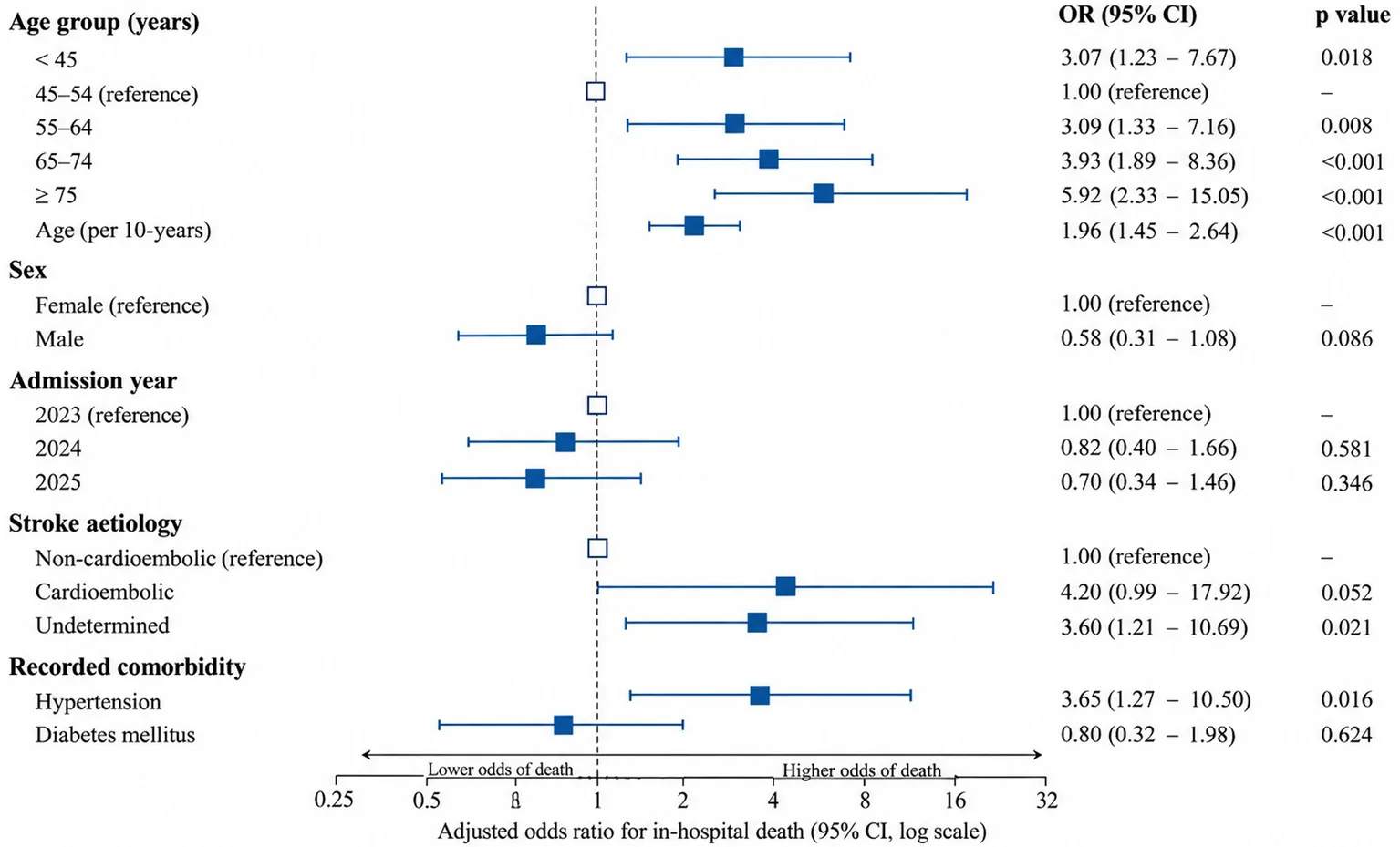
Adjusted odds ratios for in-hospital death after stroke (Model C, *n* = 479, 75 events). Multivariable logistic regression adjusted for age band, sex, admission year, stroke subtype, recorded hypertension and recorded diabetes. The reference age band is 45–54 years. Every point estimate, confidence interval and *p*-value shown here matches Table 2 and Section 3.4. Because events-per-variable is low (~7) and the smaller complete-case models showed poor calibration, the estimates are exploratory and describe associations of registry-captured variables with in-hospital death rather than independent prognostic effects.

Subgroup analyses were pre-defined at this revision: the age effect within each sex, age-band mortality and comorbidity effects within each stroke subtype, and a likelihood-ratio test of the age x subtype interaction. Sensitivity analyses re-ran the primary figures (i) after removing the single exact duplicate episode (*n* = 478), (ii) after excluding two episodes whose outcome field contained comorbidity text (*n* = 477), (iii) with the four transferred episodes counted as discharged alive (*n* = 483), and (iv) with ‘not recorded’ comorbidity treated as missing rather than negative. All analyses used Python 3 with pandas, SciPy, statsmodels and matplotlib; the complete script is provided (Supplementary material S2). The study is reported in line with the STROBE checklist for observational studies.

### 2.6 Ethical approval

The study was approved by the Hospital do Prenda Ethics Committee (reference HPDP 109/2026) and was conducted in accordance with the Declaration of Helsinki, applicable Angolan bioethics legislation and regulations, and the Angolan Personal Data Protection Law (Law No. 22/11 of 17 June). Written informed consent for participation was not required in accordance with the national legislation for retrospective analysis of de-identified routine records.

### 2.7 Data availability

The de-identified, anonymised dataset and the complete analysis code are provided as Supplementary material (S1: anonymised episode-level dataset; S2: analysis script; S3: results workbook containing the verification table, full Table 1, crude and adjusted analyses, subgroup results, data-quality audit and the comorbidity free-text inventory). Every result reported in this manuscript is reproducible from these files. Access to the non-anonymised institutional record remains subject to the Hospital do Prenda data custodian and Angolan data-protection legislation.

**Table 1:** Cohort characteristics by in-hospital vital status (*n* = 479).

Variable	Level	*n*	%	Survivors *n* (%)	Deaths *n* (%)	Mortality (%)	*p*
Sex	F	305	63.7	257 (63.6)	48 (64.0)	15.7	0.878
	M	171	35.7	145 (35.9)	26 (34.7)	15.2	
Admission year	2023	120	25.1	99 (24.5)	21 (28.0)	17.5	0.809
	2024	142	29.6	121 (30.0)	21 (28.0)	14.8	
	2025	217	45.3	184 (45.5)	33 (44.0)	15.2	
Age group (years)	<45	116	24.2	96 (23.8)	20 (26.7)	17.2	0.0049
	45–54	134	28.0	125 (30.9)	9 (12.0)	6.7	
	55–64	99	20.7	83 (20.5)	16 (21.3)	16.2	
	65–74	68	14.2	55 (13.6)	13 (17.3)	19.1	
	≥75	50	10.4	36 (8.9)	14 (18.7)	28.0	
	Age missing	12	2.5	9 (2.2)	3 (4.0)	25.0	
Stroke subtype	Ischaemic	219	45.7	184 (45.5)	35 (46.7)	16.0	0.627
	Haemorrhagic	235	49.1	201 (49.8)	34 (45.3)	14.5	
	Unspecified	11	2.3	8 (2.0)	3 (4.0)	27.3	
	Unclassified (combined)	25	5.2	19 (4.7)	6 (8.0)	24.0	
Recorded hypertension	True	95	19.8	77 (19.1)	18 (24.0)	18.9	0.324
	False	384	80.2	327 (80.9)	57 (76.0)	14.8	
Recorded diabetes	True	51	10.6	43 (10.6)	8 (10.7)	15.7	0.995
	False	428	89.4	361 (89.4)	67 (89.3)	15.7	
Head CT performed	Yes	459	95.8	390 (96.5)	69 (92.0)	15.0	0.071
	No	20	4.2	14 (3.5)	6 (8.0)	30.0	
Any comorbidity recorded	True	158	33.0	125 (30.9)	33 (44.0)	20.9	0.027
	False	321	67.0	279 (69.1)	42 (56.0)	13.1	
Readmission	Yes	13	2.7	13 (3.2)	0 (0.0)	0.0	—
Length of stay (days), median (IQR)	Survivors/decedents			6 (3–12)	3 (1–6)		<0.0001

## 3 Results

### 3.1 Cohort and data quality

483 episodes were captured across the 3 years (120 in 2023, 142 in 2024, 221 in 2025). After excluding the four transferred outcomes (all in 2025), 479 episodes entered the mortality analysis (305 women, 171 men, 3 with sex not recorded; 12 with age not recorded). The data-quality audit identified one exact duplicate episode (identical patient, dates and diagnosis), two episodes in which comorbidity text had been entered into the outcome field, and three admission-date anomalies (one unparseable date; two date pairs with an apparent year typo). Each was handled explicitly (Section 2.5, sensitivity analyses) and none changes any primary result. No episode was operated on; 13 episodes were flagged as readmissions; head CT was performed in 459/479 episodes (95.8%).

### 3.2 In-hospital mortality

75 deaths occurred: 15.7% (95% CI 12.7–19.2). Deaths were early: 44.0% within the first 24 h of admission, 58.7% by day 3 and 85.3% by day 7 (Figure 2). Median length of stay was 6 days (IQR 3–12) in survivors versus 3 days (IQR 1–6) in decedents (*p* < 0.0001, Mann–Whitney); we read this as the timing of in-hospital death, not as length of stay predicting death. Length of stay is truncated by the outcome and is reported as a descriptive timing variable only.

**Figure 2:**
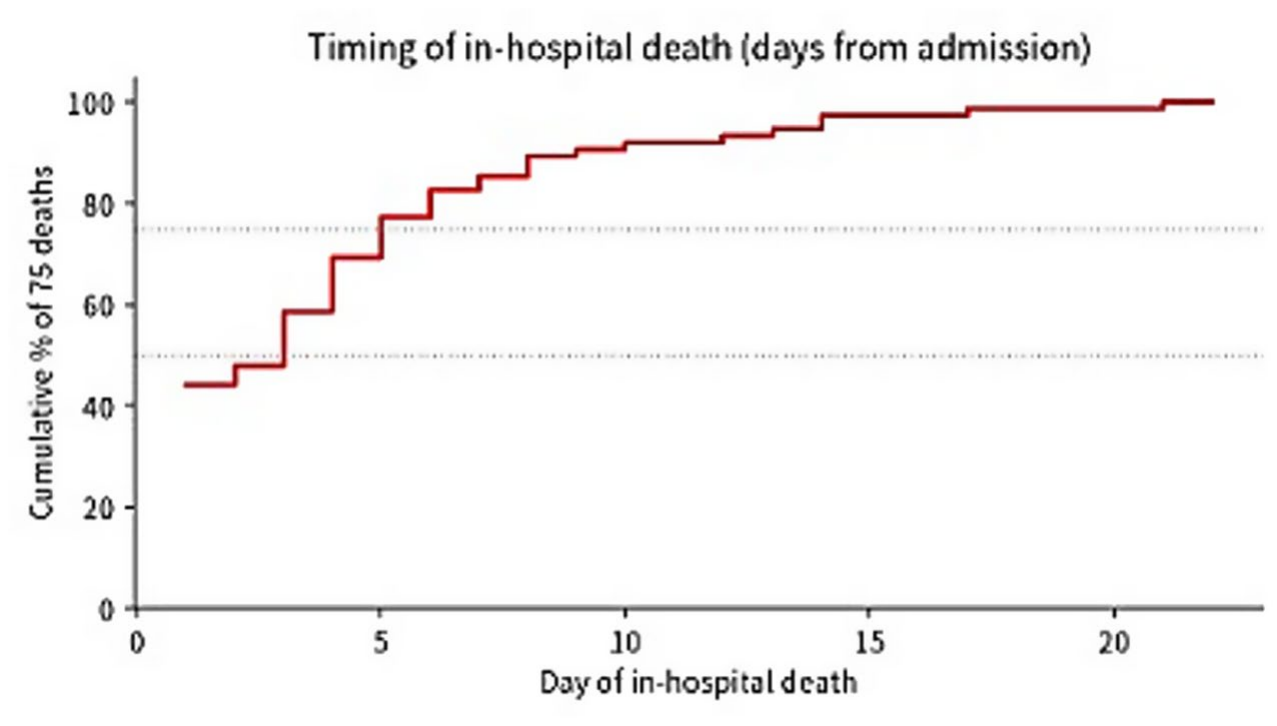
Timing of in-hospital death (75 deaths). Cumulative percentage of deaths by day of death: 44.0% within 24 h, 58.7% by day 3, 85.3% by day 7.

### 3.3 Univariable analyses

Mortality was 15.7% (48/305) in women and 15.2% (26/171) in men (crude OR 0.96, 95% CI 0.57–1.61; *p* = 0.878); three episodes with sex not recorded included one death. Mortality did not vary across years (17.5% in 2023, 14.8% in 2024, 15.2% in 2025; chi-square *p* = 0.809). Median age was 58.5 years (IQR 47–68) in decedents versus 52.0 (IQR 39–64) in survivors (*p* = 0.049). The age-band pattern was not monotonic (Figure 3, Table 1): mortality fell from 17.2% below 45 years to 6.7% in the 45–54 band, then climbed through 16.2, 19.1 and 28.0% across the older bands (overall *p* = 0.0049). The twelve episodes with no recorded age had 25.0% mortality (3/12).

**Figure 3:**
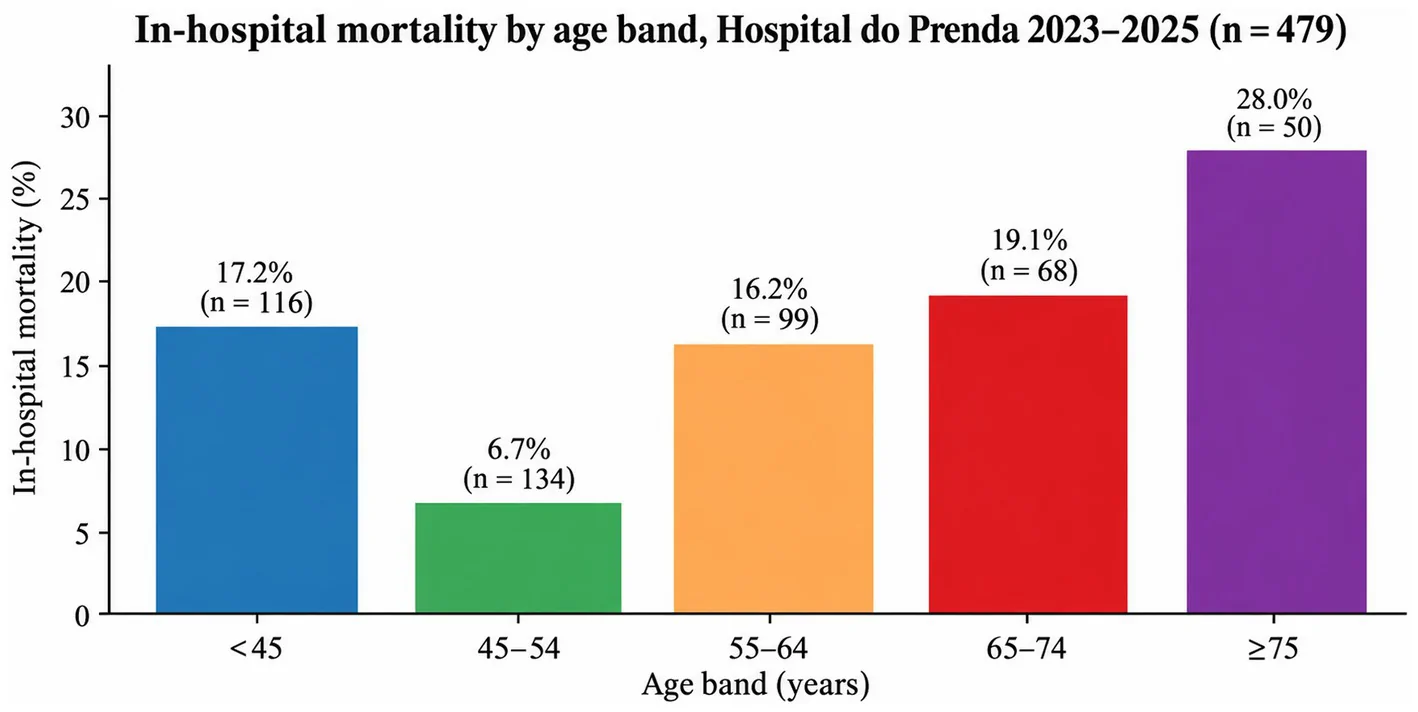
In-hospital mortality by age band (*n* = 479). Bar chart of mortality (%) with the number of episodes per band.

Mortality by subtype was 16.0% (35/219) for ischaemic, 14.5% (34/235) for haemorrhagic, and 24.0% (6/25) for the combined unclassified group; the haemorrhagic-versus-ischaemic comparison gave a crude OR of 0.89 (95% CI 0.53–1.48; *p* = 0.654). Recorded hypertension was present in 95/479 (19.8%) and was not associated with death (crude OR 1.34, 95% CI 0.75–2.41; *p* = 0.324); recorded diabetes (51/479; 10.6%) likewise showed no association (crude OR 1.00, 95% CI 0.45–2.23; *p* = 0.995). Level by level, these results are reproduced from the source data (Supplementary Table S1); the null estimates must be read against the documented under-ascertainment of the free-text comorbidity field and cannot be interpreted as evidence of absence of prognostic relevance (Section 4.4).

Two additional registry descriptors were analysed for the first time. Episodes without head CT had substantially higher mortality than those with CT (6/20, 30.0%, versus 69/459, 15.0%; crude OR 2.42, 95% CI 0.90–6.52; *p* = 0.071). Episodes with any comorbidity recorded had higher mortality than those without (33/158, 20.9%, versus 42/321, 13.1%; crude OR 1.75, 95% CI 1.06–2.90; *p* = 0.027). Both are interpreted as ascertainment-related signals, not biological effects (Section 4.3).

### 3.4 Adjusted analyses

In the complete-case adjusted model (Model B, *n* = 467, 72 events) no covariate reached statistical significance, including age (adjusted OR 1.18 per 10 years, 95% CI 0.99–1.41; *p* = 0.069), recorded hypertension (1.64, 0.87–3.09; *p* = 0.124) and recorded diabetes (0.89, 0.39–2.05; *p* = 0.786) (Table 2). When age was entered as bands on the full cohort (Model C, *n* = 479, 75 events), the age gradient was strong and graded relative to 45–54 years: adjusted OR 2.97 (95% CI 1.28–6.87, *p* = 0.011) for age <45; 2.90 (1.20–7.01, *p* = 0.018) for 55–64; 3.59 (1.42–9.05, *p* = 0.007) for 65–74; and 5.92 (2.28–15.38, *p* = 0.0003) for age ≥75; the missing-age category had adjusted OR 3.86 (0.83–17.96, *p* = 0.085). Model C reached a c-statistic of 0.649 (Hosmer-Lemeshow *p* = 0.797). Figure 1 displays the Model C estimates as a forest plot; Adding CT status (Model A) left the age effect essentially unchanged (age adjusted OR 1.16 per 10 years, 95% CI 0.97–1.39); the CT coefficient itself could not be estimated stably (quasi-separation with only 20 CT-negative episodes) and is reported only descriptively (Section 3.3). Because events-per-variable is low (~7) and the Hosmer–Lemeshow test was significant in the smaller complete-case models (*p* = 0.003), all adjusted estimates should be treated as exploratory and associative; they describe how registry-captured variables co-vary with in-hospital death, and should not be interpreted as estimates of an independent prognostic effect (Section 4.1).

**Table 2:** Crude and adjusted odds ratios for in-hospital death (adjusted estimates from Model C, *n* = 479, 75 events; 45–54 y is the reference age band).

Term	Crude OR (95% CI)	*p*	Adjusted OR (95% CI)	*p*
Age, per 10 years (Model B)	1.18 (1.00–1.40)	0.052	1.18 (0.99–1.41)	0.068
Age < 45 (vs 45–54)	—	—	2.97 (1.28–6.87)	0.011
Age 55–64 (vs 45–54)	—	—	2.90 (1.20–7.01)	0.018
Age 65–74 (vs 45–54)	—	—	3.59 (1.42–9.05)	0.007
Age ≥ 75 (vs 45–54)	—	—	5.92 (2.28–15.38)	<0.001
Age not recorded (vs 45–54)	—	—	3.86 (0.83–17.96)	0.085
Male sex (vs female)	—	—	0.95 (0.55–1.64)	0.844
Admission year 2024 (vs 2023)	—	—	0.82 (0.40–1.72)	0.604
Admission year 2025 (vs 2023)	—	—	0.78 (0.38–1.58)	0.485
Haemorrhagic (vs ischaemic)	0.89 (0.53–1.48)	0.654	0.79 (0.44–1.42)	0.439
Unclassified (vs ischaemic)	—	—	2.03 (0.50–8.31)	0.326
Recorded hypertension	1.34 (0.75–2.41)	0.324	1.64 (0.87–3.09)	0.124
Recorded diabetes	1.00 (0.45–2.23)	0.995	0.89 (0.39–2.05)	0.786
Head CT not performed	2.42 (0.90–6.52)	0.071	Not estimated (quasi-separation)	-

Reference categories: age 45–54 years; female sex; 2023; ischaemic stroke; recorded comorbidity absent; head CT performed. Adjusted estimates for the age bands, sex, admission year and stroke subtype come from model C; the per-10-years age estimate comes from the complete-case model B for consistency. All adjusted estimates are exploratory and associative, not estimates of independent prognostic effect.

### 3.5 Subgroup and interaction analyses

The age effect appeared driven by men: per-10-year crude OR 1.40 (95% CI 1.04–1.89, *p* = 0.026) in men versus 1.05 (95% CI 0.85–1.30, *p* = 0.622) in women. The age pattern was present within both ischaemic and haemorrhagic strokes, but the likelihood-ratio test of the age × subtype interaction was significant (*p* = 0.0009). The elevated mortality of the youngest patients was concentrated among those with haemorrhagic stroke: among patients younger than 45 years, 16/72 haemorrhagic episodes (22.2%) ended in death versus 4/37 ischaemic episodes (10.8%) (Table 3). Subgroup cell counts are small, so both the sex-stratified and the age-by-subtype comparisons should be interpreted with corresponding caution.

**Table 3:** Subgroup and interaction analyses.

Subgroup	*n* (deaths)	Mortality %	Estimate (95% CI)	*p*
Age effect, women (per 10 years)	297 (45)	15.2	OR 1.05 (0.85–1.30)	0.622
Age effect, men (per 10 years)	168 (26)	15.5	OR 1.40 (1.04–1.89)	0.026
Ischaemic: age-band mortality	219 (35)	16.0	<45: 10.8%; 45–54: 8.0%; 55–64: 13.5%; 65–74: 24.3%; ≥75: 28.9%	—
Haemorrhagic: age-band mortality	235 (34)	14.5	<45: 22.2%; 45–54: 3.9%; 55–64: 20.5%; 65–74: 7.1%; ≥75: 11.1%	—
Age < 45, haemorrhagic episodes	72 (16)	22.2	—	—
Age < 45, ischaemic episodes	37 (4)	10.8	—	—
Interaction: age × subtype	454	—	Likelihood-ratio test	0.0009

Age bands: <45; 45–54; 55–64; 65–74; ≥75 years. Estimates are crude. Cell counts in some cells are small; findings are descriptive.

## 4 Discussion

### 4.1 Interpretation of the age gradient

The central finding of this study is that older age is the only characteristic strongly and consistently associated with in-hospital death, with a graded association from 6.7% (45–54 years) to 28.0% (≥75 years) in crude analysis (*p* = 0.0049) and adjusted odds ratios rising to 5.92 (95% CI 2.28–15.38) for the oldest band in Model C. The direction replicates the Weimar derivation (6) and the Ghanaian and Mozambican series (2, 3). The absolute levels are far above what international scores would predict from age alone, consistent with a catchment where median onset-to-door time exceeds 14 h (11): late-arriving, untreated cohorts accumulate the early deaths that stroke units elsewhere prevent.

The adjusted age-band estimates are drawn from a model with approximately seven events per variable and, in the smaller complete-case models, evidence of poor calibration (Section 2.5). They should be read as descriptive of the association between age and in-hospital death in this registry, not as estimates of an independent prognostic effect of age holding admission severity, pre-stroke function and acute treatment constant. None of those constructs is captured in the registry.

### 4.2 Younger patients and haemorrhagic stroke

The non-monotonic pattern - 17.2% mortality in patients younger than 45, above the 6.7% of the 45–54 band - admits two interpretations. One is that younger Angolan stroke patients present with more severe syndromes - a heavier haemorrhagic load, more malignant middle-cerebral-artery events - consistent with what Goncalves and Ricardo noted in the same city (11), but which the registry cannot adjudicate because admission imaging was not consistently subtyped and admission severity is not recorded. The other is a denominator effect: the <45 band is small, and a handful of very-early deaths lifts the proportion. The elevated mortality persisted after adjustment (Model C adjusted OR 2.97, 95% CI 1.28–6.87) and the age × subtype interaction was significant (*p* = 0.0009): 22.2% of haemorrhagic episodes in the youngest band ended in death versus 10.8% of ischaemic ones. In the absence of stroke-severity data and given the small denominators, this pattern is described as elevated mortality concentrated among patients with haemorrhagic stroke; we do not claim that haemorrhage caused the excess death, only that it is the subtype in which the excess is concentrated in this cohort.

### 4.3 The haemorrhagic-versus-ischaemic null

The overall subtype comparison returned 14.5% versus 16.0% (crude OR 0.89, 0.53–1.48), against the substantial haemorrhagic excess reported internationally (13, 14). Two structural explanations remain. First, the unclassified category - 25 episodes, 24.0% mortality - almost certainly absorbs more haemorrhagic than ischaemic cases, since the most severe haemorrhagic patients in this catchment often die before reaching CT and are never subtyped; their deaths are counted against the unclassified denominator, raising it, while removing the highest-mortality events from the haemorrhagic denominator. Second, the CT-negative group (4.2% of episodes) had double the mortality of the CT-positive group (30.0% vs. 15.0%), consistent with CT being withheld from the most severe or most rapidly fatal patients. Both mechanisms compress the apparent subtype gradient and are consistent with ascertainment, not biology, driving the null.

### 4.4 Hypertension and diabetes: ascertainment, not absence of effect

The crude and adjusted nulls for hypertension (crude OR 1.34, 95% CI 0.75–2.41; adjusted OR 1.64, 0.87–3.09) and diabetes (crude OR 1.00, 0.45–2.23; adjusted OR 0.89, 0.39–2.05) must not be read as evidence that these comorbidities are unimportant. Recorded hypertension reached only 19.8% of the cohort, against the ~88% case prevalence measured by INTERSTROKE Angola (12) - roughly four of five hypertensive patients in this catchment carry no recorded comorbidity in the Hospital do Prenda registry. The comorbidity field is a single free-text line completed unevenly across admissions, and misclassification of this kind is expected to bias the observed associations toward the null. The observed association between any recorded comorbidity and death (crude OR 1.75, 95% CI 1.06–2.90) is consistent with an ascertainment effect - the field tends to be completed for the more investigated or more severe patients - and is reported as such, not as a biological claim. The hypertension and diabetes estimates should therefore be interpreted with caution and not as evidence of absence of prognostic relevance.

### 4.5 Timing of death

Death occurred early and fast: 44.0% within 24 h, 58.7% by day 3, 85.3% by day 7. This timing matches the upstream Luanda presentation pattern (median onset-to-door >14 h; only 10.8% arriving within the thrombolysis window) (11) and the INTERSTROKE Angola finding that only 27.4% of cases reach a Glasgow Outcome Scale of 5 at discharge (12). When patients arrive late and no intensive-care escalation is available, mortality concentrates in the first days.

### 4.6 Limitations

The registry does not record admission neurological severity (NIHSS, GCS), pre-stroke functional status (mRS), admission glucose, inflammatory markers or imaging features beyond the broad subtype label; it does not record ICU admission, mechanical ventilation or thrombolysis use in a usable form (15). The absence of these key prognostic variables represents substantial residual confounding, and the reported associations should be interpreted strictly as associations - the study cannot estimate the independent prognostic effect of any variable holding admission severity, pre-stroke function and acute treatment constant. Comorbidities come from a single free-text field with documented under-ascertainment, biasing comorbidity associations toward the null. Adjusted models carry approximately 7 events per variable and the Hosmer–Lemeshow test was significant in the smaller complete-case models (*p* = 0.003), so calibration is imperfect; all adjusted estimates - including the age gradient and the subgroup and interaction findings - are therefore exploratory. Subgroup denominators, in particular for patients younger than 45 years and for the unclassified stroke category, are small; the age × subtype interaction and the sex-stratified age effect should be regarded as hypothesis-generating. The cohort is single-centre and the sample size was fixed by the registry, so no a-priori power calculation applies.

## 5 Conclusion

Older age was the only admission-available characteristic robustly associated with in-hospital death after stroke at Hospital do Prenda, with a graded adjusted gradient, and the elevated mortality of the youngest patients was concentrated among patients with haemorrhagic stroke. Recorded comorbidity is poorly captured, so the null estimates for hypertension and diabetes should not be read as evidence of absence of prognostic relevance. In the absence of stroke-severity, pre-stroke functional status, ICU/mechanical-ventilation and reperfusion data, all findings are reported as associations of registry-captured variables with in-hospital death, not as estimates of independent prognostic effect. The anonymised dataset and complete analysis are provided so that the results can be independently verified. Structural reform - routine documentation of admission severity, structured coding of comorbidities and a national Angolan stroke registry harmonised with sub-Saharan neighbours - remains overdue.

## Data Availability

The raw data supporting the conclusions of this article will be made available by the authors, without undue reservation.
